# Supplementary material for: Atomic scale insights into NaCl nucleation in nanoconfined environments
Source: Chem Sci. 2024 Aug 23;15(37):15391–8. doi: 10.1039/d4sc04042b (PMC11367593; doi:10.1039/d4sc04042b)
Supplement: SC-015-D4SC04042B-s001 [file SC-015-D4SC04042B-s001.pdf]

Supplementary Information (SI) for Chemical Science.
This journal is © The Royal Society of Chemistry 2024

Supplementary material: Atomic scale insights into NaCl nucleation in nanoconfined environments

Ruiyu Wang[1] and Pratyush Tiwary[1,2,3,*]

[1] *Institute for Physical Science and Technology, University of Maryland, College Park, MD 20742, USA*

[2] *Department of Chemistry and Biochemistry, University of Maryland, College Park, MD 20742, USA*

[3] *University of Maryland Institute for Health Computing, Bethesda, Maryland 20852, USA*

[*] *ptiwary@umd.edu*

I. ADDITIONAL DETAILS OF MD SIMULATIONS AND CALCULATIONS

A. State predictive information bottleneck

The state predictive information bottleneck (SPIB) is a deep learning method to extract RCs from MD simulations using a variational autoencoder. The principle, implementation, and application of SPIB are discussed in previous papers [S1–S4]. The encoder type is either linear or nonlinear. When using a linear encoder, SPIB learns a linear combination of input OPs as the reaction corrdinates because it has reproduced the committor [S5, S6]. Such RC can also be used in enhanced sampling simulations such as well-tempered metadynamics (WTmetaD). A nonlinear encoder has a higher capability to distinguish metastable states, such as liquid, solid, and other states. Therefore, it is used to analyze the final production run of the simulations.

B. Protocol to run enhanced sampling using SPIB

The protocol to run metadynamics using SPIB has been discussed in previous papers.[S3] Here, a summary of the procedure is listed:

1. Run WTMetaD simulations by biasing a simple, human-based trial RC, a linear combination of $\overline{N}$ and U_E, marked as *SPIB0*.

2. Repeat the following two steps twice:

   - Collect the trajectories of OPs obtained from simulations from the last step. Run SPIB with standardized selected OPs using a linear encoder to generate RCs as a linear combination of OPs, denoted as *SPIBx*, where x is how many times SPIB has been used.

   - Run MetaD simulation using the RC *SPIB1* and *SPIB2*. Simulations using RC *SPIB2* are based on WTMetaD and they are used for final analysis, such as OP importance evaluation and free energy calculation.

These OPs are selected in metadynamics: $\overline{q_4}, \overline{q_6}, U_E, \overline{N}, \mu_N^2, N_{2+}, N_{3+}, N_{4+}, N_{5+}, \overline{N_W}$ and N_{W1-}. The OP N_{5+} is not included in simulations for $d = 0.8$ nm because ions under such confinement are in a single layer and can only pair with 4 counterions (Fig. S1 and

S2). In this work, we use SPIB to learn 2d RCs since it is difficult to sample along 3 or even higher-dimensional RCs.

To run enhanced sampling MD simulations, in most simulations, WTMetaD was applied except the *SPIB1* in simulations for d = 1.9 and 3.9 nm, in which transition-tempered metadynamics (TTMetaD) was applied to reduce the simulation time. The principle and application of TTMetaD have been discussed in previous papers [S7, S8].

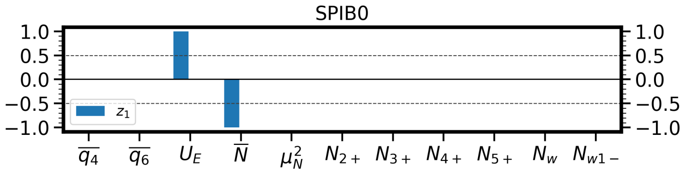

FIG. S1. Scaled standardized coefficients of *SPIB0*, which is the same for all four thickness (d = 0.8, 1.2, 1.9 and 3.9 nm). For clarity, the highest weight on these OPs is set as 1 in the figure.

C. Simulation settings of single ion pairing

To calculate the free energy surface (FES) of a single ion pairing, we modify the simulation settings for nucleation. We only keep one NaCl molecule and replace other NaCl with waters. The distance between the two ions (r) is biased for WTMetaD using PLUMED [S9]. Bias potential, 1 kJ/mol with a σ of 0.02 nm, is added every 4 ps. The bias factor is 30. The upper limit of r is set as 1.6 nm. We also run simulations with additional thickness: d = 0.9, 1.0, and 1.1 nm. Their settings are listed below (Table S1).

TABLE S1. Other parameters of additional simulations d = 0.9 to 1.1 nm. The unit of thickness is nm.

Thickness (d)	Water
0.90 (d=0.9)	448
1.00 (d=1.0)	598
1.12 (d=1.1)	698

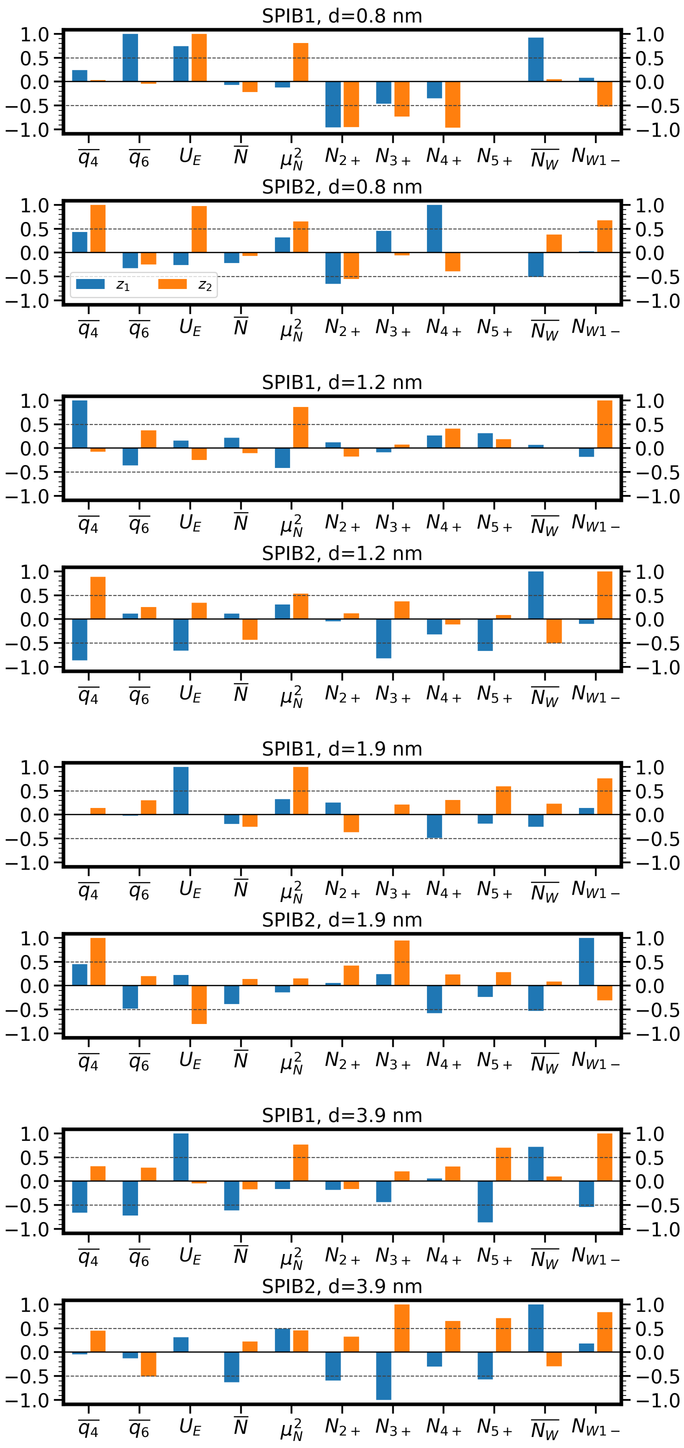

FIG. S2. Scaled standardized coefficients of all simulations in this work, in which the weight of the highest OP is set as 1. *SPIB1* and *SPIB2* represent how many times the RCs are learned from SPIB as discussed above. "z_1" (blue) and "z_2" (yellow) are the two components in the RCs.

D. Free energy calculation and reweighted histogram

To calculate the free energy surface and relative stability of phase states or single ion pairing from WTMetaD simulations, the reweighted histogram method, which has been discussed in a previous paper[S10], is applied. In principle, In biased MD simulations, the weight of every configuration sampled is calculated by:

$$w(t) = e^{\beta(V(s,t)-c(t))} \tag{S1}$$

where $w(t)$ is the weight of the configuration at time t; s is the RC; V is the bias potential added and $c(t)$ is the reweighting factor calculated by:

$$e^{\beta c(t)} = \int ds[e^{\gamma V(s,t+\Delta t)/k_\mathrm{B}\Delta T} - e^{\gamma V(s,t)/k_\mathrm{B}\Delta T}], \tag{S2}$$

where $\beta = 1/k_B T$, γ is the bias factor, and the free energy surface $F(s)$ is calculated by:

$$F(s) = -\frac{1}{\beta} log \iint w(t)\delta(s' - s)dtds'. \tag{S3}$$

The free energy for phase transition is calculated by:

$$\Delta A_{L-S} = \mu_S - \mu_L = -\frac{1}{\beta} log(P_S/P_L), \tag{S4}$$

P_L and P_S are the Boltzmann probabilities of liquid and solid states calculated by reweighting simulations using Eq. S1. The states are identified using Nonlinear SPIB, which is discussed in section *Free energy surfaces using SPIB RCs*

E. OP relevance evaluation

Similar to previous paper, the Thermodynamically Explainable Representations (TERP) is applied to measure the significance of OPs as discussed in the main text [S2, S11, S12]. The details of the principle and implementation of TERP have been discussed in other papers [S12]. TERP analysis does not rely on time series data and we pick configurations near the transition state of liquid-solid phase transition to reduce the errors caused by OPs that correlate but not drive the phase transition. In addition, TERP adds a penalty to the number of selected features to exclude insignificant OPs. The procedure to carry out TERP calculation is listed below:

1. Run SPIB calculation using nonlinear encoders to trajectories obtained from WT-MetaD simulations using the RC SPIB2.

2. Identify the points at the transition state. If the probability of solid and liquid predicted by SPIB are both higher than 0.49, 5000 points are randomly selected as the transition state for TERP calculation. If there are not enough points, the cutoff of probability is reduced to 0.48, and 500 points are selected.

3. Perform TERP using the points selected in the previous step and SPIB nonlinear model in step 1.

II. ADDITIONAL RESULTS AND DISCUSSIONS

A. Water layering and ion pairing from unbiased simulations

The structure and dynamics of waters and ions should not be ignored when investigating chemical and physical processes near aqueous interfaces [S13]. We carried out unbiased MD simulations from the same initial configurations for WTMetaD. Except for $d = 0.8$ nm, we hardly observe any phase transitions and ions stay in the liquid state. Near the graphene surfaces, there is a pronounced water layer, whose water density is several times higher than that of bulk water at room temperature (about 33 nm^{-1}). In simulations with pure waters without ions, these water layers have high $\epsilon_{\parallel}$ [S14]. Interestingly, the concentration of ions at the interfaces also differs from the bulk: ions tend to stay away from the graphene interfaces. It is consistent with previous observation that small ions, whose polarization is rather weak, do not adsorb to hydrophobic interfaces [S15, S16]. Although the high density water layers remove ions from the high dielectric constant regions, between each water layer, the local concentration of ions are even higher than that of the bulk. For $d = 3.9$ nm, with the thickest water slab in this work, the density of water and ions shows no peak and recovers to the bulk-like structures at the center of the water slab with a size of 1 nm. The bulk number density of ions is about 5 nm^{-3} (Fig. S3d). For $d < 2$ nm, water slabs are too thin for nano-confined water and ions to recover the bulk-like behavior. Ion density also shows layering-like and the local densities in those peaks (about 10 nm^{-3}) are higher than that of the bulk. We believe that such water ion alternating layers promote the nucleation of NaCl for two

reasons: first, higher ion concentration prefers to be the solid phase; and, high ion density further decreases ϵ of the water slab [S17], strengthening the electric interactions between ions to form the solid phase. The ion-water radial distribution function $g(r)$ (Fig. S4) shows that the positions of peaks and basins of $g(r)$ do not change at different thicknesses d. The positions of the first basin of Na-O and Cl-O are 0.321 and 0.402 nm, respectively. Based on the result, these numbers are used as the cutoff value for ion-water coordination to calculate ion-water OPs. Other thermodynamics properties of the nanoconfined liquid, such as the pressure tensor, may also be different from that in the bulk.

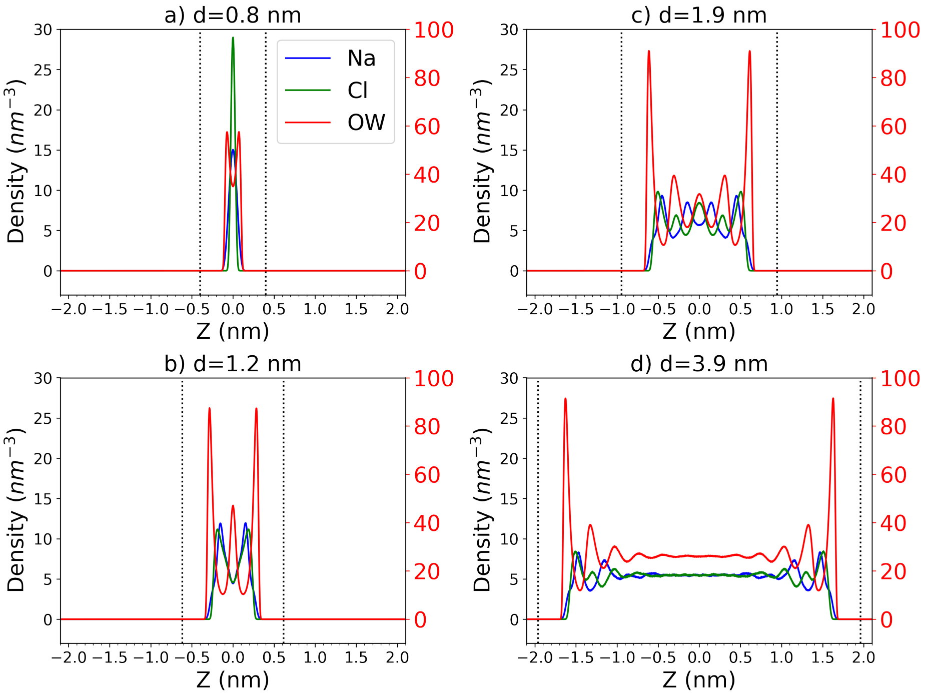

FIG. S3. Density profile of ions and waters perpendicular to graphene sheets. Water is represented using its oxygen atom. The 0 nm on the X axis is the center of two graphene sheets. Numbers on the left (black) and right (red) Y axis are for ions and waters, respectively. Dot lines are the positions of graphene sheets.

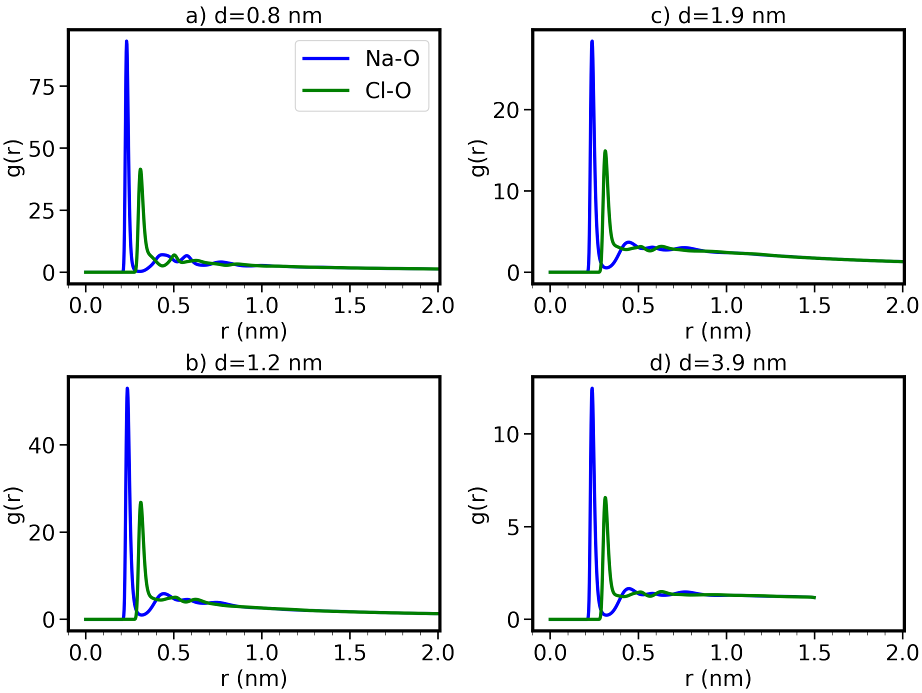

FIG. S4. Ion-water radial distribution function $g(r)$.

B. Crystal structures and relative stability under confinement

1. *Free energy surfaces using SPIB RCs*

The free energy surface (FES) calculated using the reweighted histogram method[S10] is continuous for the 4 thicknesses in this work (Fig. S5 a-d). Combining with the metastable states identified by SPIB (Fig. S5 e-h), there are small observable barriers for the transition between liquid and solid, either less than 25 kJ/mol ($d = 0.8$ and 1.9 nm) or between 25 and 50 KJ/mol ($d = 1.2$ and 3.9 nm). However, unbiased simulations cannot overcome these barriers, indicating the existence of hidden barriers [S11]. There is no observable barrier for the liquid-hydrated-phase transition. As discussed above, we have built 2d RCs as a compromise of complexity and sampling efficiency. The FES shows that RCs do not fully take advantage of the two dimensions. For $d = 0.8$ nm, both z_1 and z_2 are sampled and SPIB distinguishes metastable states based on z_1, in other words, z_1 may be enough to distinguish liquid and solid. At other thicknesses, sampling is along the diagonal of the two components of RCs. Further reducing to a 1d RC as a linear combination of z_1 and z_2 is possible, but the width of the FES shows that there is still some information in the 2d RCs. Finally,

although SPIB using nonlinear encoder has a larger capability of data compression and a better metastable state identification is expected, states predicted generally follow the trend of linear SPIB (Fig. S5). The boundary between states is either based on z_1 ($d = 0.8$ nm) or follows the diagonal (at other thicknesses).

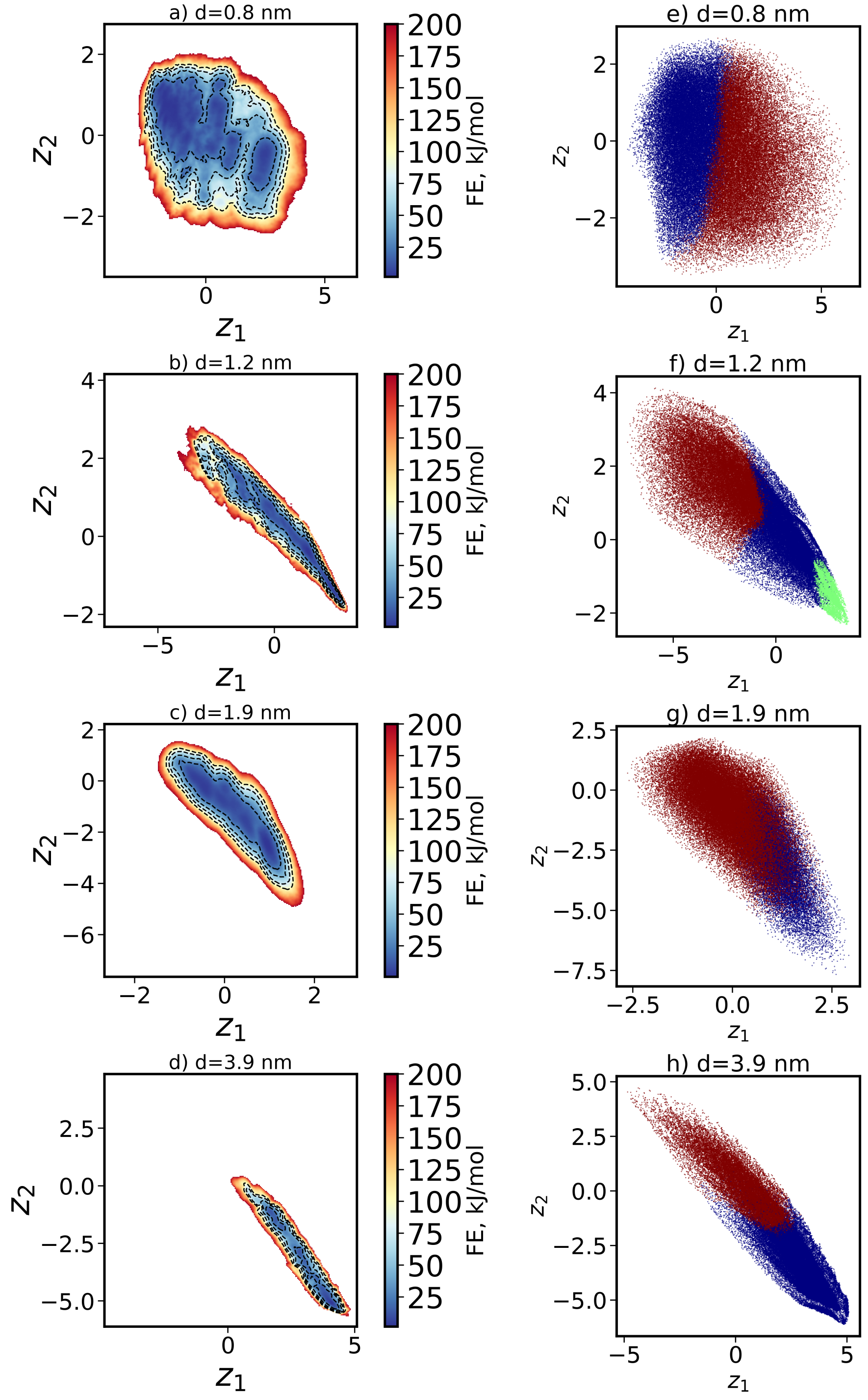

FIG. S5. Free energy surfaces (FES) and phase states identified by SPIB. a) - d) FES plotted using SPIB learned RCs z_1 and z_2. Grids that with free energy higher than 200 kJ/mol are not shown. The gradient of contour lines are 25 kJ/mol per line with an upper limit of 100 kJ/mol. e) - h) States identified by SPIB. Red, blue, and green points represent solid, liquid, and hydrated states.

2. Free energy surfaces using other possible RCs

The advantage of machine learning RCs is that they can be biased in WTMetaD simulations and they can distinguish those metastable states. However, the RCs are absurd to connect to straightforward physical properties of simulations. In the case of nucleation, the OP, numbers of ions belonging to the FCC solid, has been introduced and proven to be efficient to describe nucleation processes [S18]. Since the nucleation of NaCl follows the one-step mechanism, the number of FCC ions can be represented by number of ions within the largest cluster (C_M), which is much easier to compute [S19]. Otherwise, the existence of large disordered clusters is evidence of the two-step mechanism.

As per the classical nucleation theory (CNT), the free energy with C_M keeps increasing before the critical nuclei size and then starts to decrease. However, there are several challenges in MD simulations to reproduce the trend. First, as in classical MD simulations, the selection of force fields determines the properties of ions but they may not reproduce the experimental saturated concentration c_s. For $d = 3.9$ nm here and bulk solution in previous paper [S11], simulations predict a free energy increase with the growth of the size of solid phase and liquid is more stable than the solid phase, indicating that the force field in this work predicts that $1.5c_s$ may not be high enough to turn liquid NaCl solution to the solid. In addition, due to the finite size effect, depletion during the growth of solid makes it difficult to observe a big crystal and estimate the size of critical nuclei. To solve the problem, constant chemical potential MD (CμMD) that controls the concentration of the solution is needed [S19].

Our results show that the free energy keeps increasing as C_M increases. For $d = 0.8$ nm, the free energy grows evenly from $C_M = 20$ to 100. What is more, the free energy for $C_M = 100$ is still less than 200 kJ/mol, indicating that configurations that all ions are connected have been observed. At this time, the concentration of the solution is 0 and completely depleted. We believe that the strong capability of the solid phase at $d = 0.8$ nm to resist depletion is consistent with the conclusion that nano-confined environment stabilizes the solid phase. Connected to dielectric behaviors discussed in the main text, we hypothesize that under strong nano-confinement, ion-ion electric interaction is enhanced to stabilize the solid phase. Using C_M as the OP, the free energy surfaces are similar for $d = 1.2$ and 1.9 nm. Depletion prevents the formation of a large cluster [S11, S19]. However for

d = 1.2 nm, the quick rise of free energy due to depletion starts when $C_M = 150$, higher than 120 for $d = 3.9$ nm and the total number of simulations in $d = 3.9$ is larger than that in d = 1.2 nm, indicating a strong capacity for small d to resist depletion. For d = 1.9 nm, the FES shows a flat region until $C_M = 300$, consistent with the fact that liquid and solid phases have a similar stability and relative free energy, as discussed in the main text (Fig. 3a). In summary, all free energy plots with C_M demonstrate increased stability of large clusters and capability to resist depletion in solutions under nano-confinement. Based on the assumption of the one-step mechanism that large clusters belong to ordered solid, nano-confined environments facilitate the solid structures.

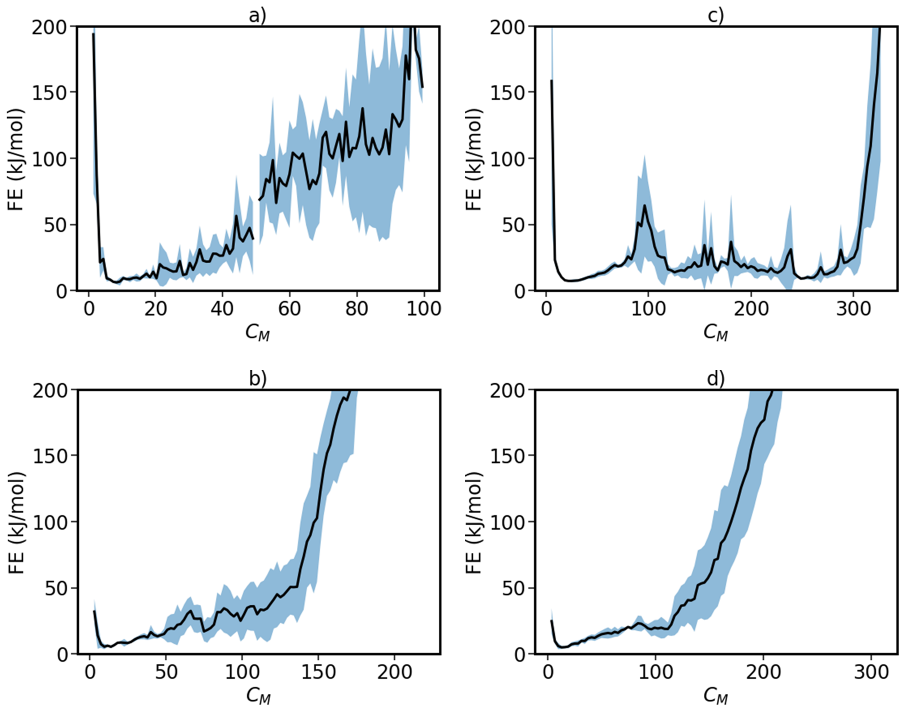

FIG. S6. Free energy surfaces using the size of the largest cluster, C_M. a) - d) represent simulations of with different thicknesses ($d = 0.8, 1.2, 1.9$, and 3.9 nm), respectively. Only free energy less than 200 kJ/mol is shown. The total numbers of ions in each thickness are 100, 220, 412, and 312.

C. Additional results of single ion pairing

As discussed in the main text, the behavior of single-ion pairing can be used to estimate the phase transition at different thicknesses. Its FES shows similar trends for $d = 0.9$ to 1.2

nm but significant disparities for $d = 0.8$ nm (Fig. S7). We attribute such observations to the effect of water layering. There are two water layers and one ion layer for $d = 0.8$ nm, but 3 water layers and 2 ion layers for $d = 1.2$ nm (Fig. S3).

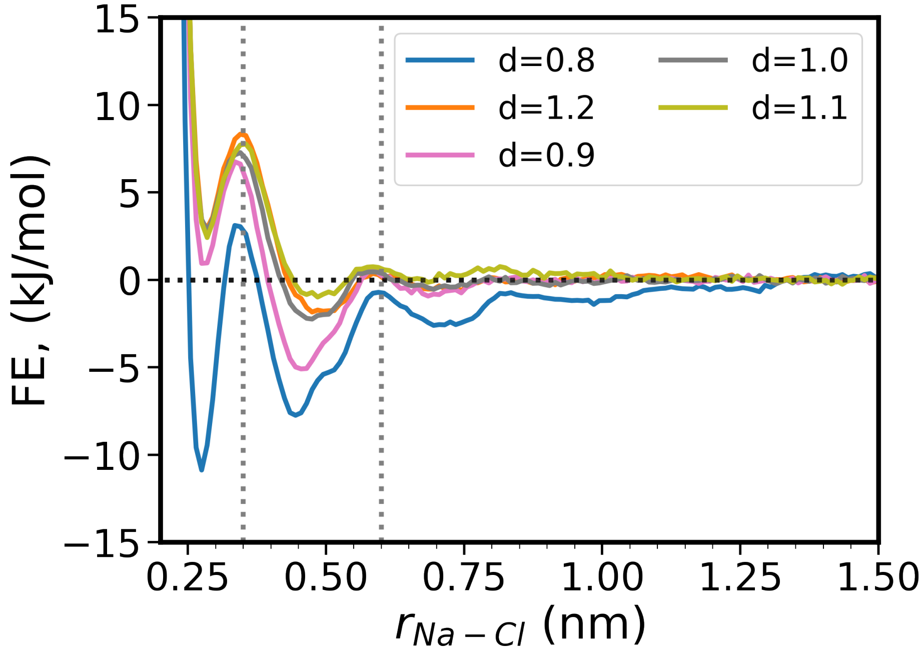

FIG. S7. FES of single ion dissociation for simulations with thickness d between 0.8 and 1.2 nm. Two dot lines represent the boundary of contact pair (CP) and solvent separated ion pair (SSIP) at $r = 0.35$ and 0.6 nm.

D. OP correlations

One challenge in machine learning techniques (both SPIB and TERP) in this work is the correlation of OPs, especially the multicollinearity that may lead to errors when calculating their coefficients using a linear model. Using the OPs collected from WTMetaD simulations, we calculated the absolute value of pair-wise correlations (Fig. S8) by:

$$r_{ij} = \frac{|C_{ij}|}{\sqrt{C_i C_j}}, \tag{S5}$$

where C is the covariance matrix. Low and high r_{ij} values represent nonlinear and linear correlations between OP i and j. Results show that U_E and CN based OPs are highly correlated except μ_N^2. Fortunately, such high correlation has been observed in bulk solutions

but SPIB and TERP can still work, demonstrating the robustness of the machine learning method used in this work [S11].

The category of electric force is different from other groups. The total forces $|F_i|$ is to some degree correlated with CN OPs, but its correlation coefficients with $|F_{ii}|$ or $|F_{iW}|$ is much lower. At the same time, $|F_{ii}|$ and $|F_{iW}|$ are highly correlated. Probably they contain different information, even though for each ion, $\boldsymbol{F}_i = \boldsymbol{F}_{ii} + \boldsymbol{F}_{iW}$ (note: $\boldsymbol{F}$ is the force vector and $|F|$ is the norm, the scalar value of the force).

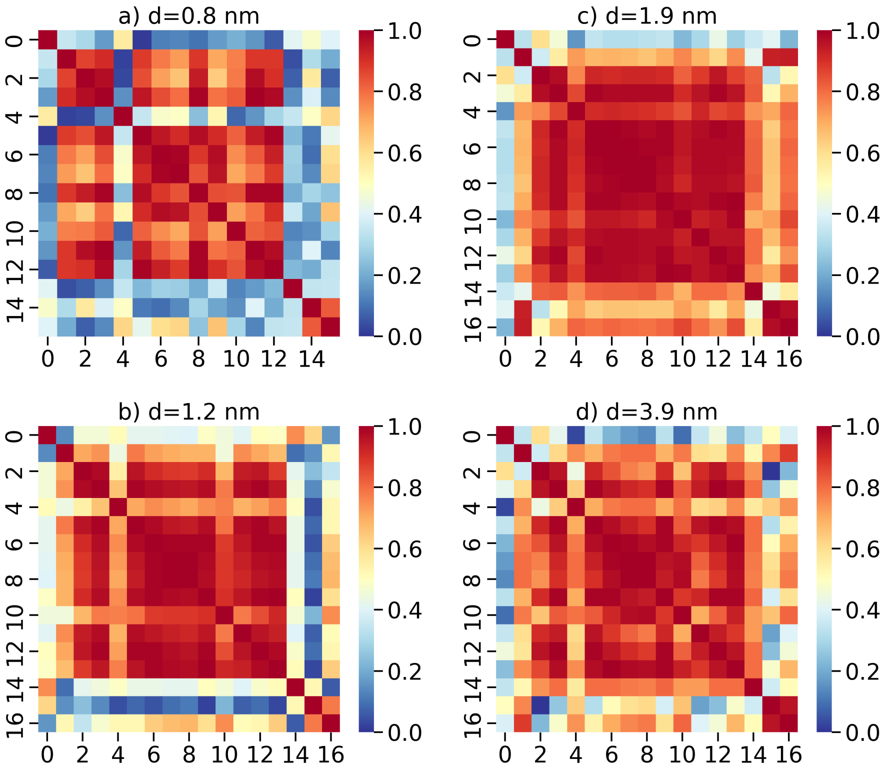

FIG. S8. Correlation coefficients (defined in Eq. S5) in simulations of each thickness. X and Y labels are the index of OPs and the associated OPs are described in Tabel S2.

TABLE S2. Indices for different OPs in Fig. S8

	0	1	2	3	4	5	6	7	8	9	10	11	12	13	14	15	16
$d = 0.8$ nm	$\overline{q_4}$	$\overline{q_6}$	U_E	$\overline{N}$	μ_N^2	N_{2+}	N_{3+}	N_{4+}	$\overline{N_w}$	N_{w1-}	C_M	$\overline{N_{NaO}}$	$\overline{N_{ClO}}$	$\overline{\lvert F_i\rvert}$	$\overline{\lvert F_{ii}\rvert}$	$\overline{\lvert F_{iw}\rvert}$	
$d > 1$ nm	$\overline{q_4}$	$\overline{q_6}$	U_E	$\overline{N}$	μ_N^2	N_{2+}	N_{3+}	N_{4+}	N_{5+}	$\overline{N_w}$	N_{w1-}	C_M	$\overline{N_{NaO}}$	$\overline{N_{ClO}}$	$\overline{\lvert F_i\rvert}$	$\overline{\lvert F_{ii}\rvert}$	$\overline{\lvert F_{iw}\rvert}$

We plotted several pairs of OPs to visualize their correlations, including N_{5+} vs. N_{W1-}

(Fig. S9), C_M vs. N_{5+} (Fig. S10), $|F_{iW}|$ vs. C_M (Fig. S11), and $|F_{iW}|$ vs. N_{W1-} (Fig. S12). In these figures, the OPs are standardized as calculated in SPIB and TERP (marked as OP name with prime and standardization does not affect their correlations). The correlation of each pair justifies some assumptions we made in this work. For N_{5+} vs. N_{W1-}, it shows that the total CN or an ion does not change too many during nucleation. The growth of nucleus requires the formation of ions with high CN with counterions by removing their last coordinated water, which is highly possible that these ions are at the surface of the previous nucleus. For C_M vs. N_{5+}, as the size of ion cluster increases, more ions become the core of the solid phase. For $|F_{iW}|$ vs. both C_M and N_{W1-}, we found that the the average norm of forces between ion and water are high in the liquid phase and low in solid phase. There may be two possible reasons: first, the ion-water forces tend to drive the dissolution of ions. Ions under a larger force are relatively unstable in the solid. The second reason is just because ions inside the cluster are far away from water and therefore cannot feel the forces from water. Last, it is worth mentioning that correlated OPs are not completely equivalent in the roles of nucleation. For instance, enthalpy and $\overline{N}$ are correlated in the trajectory of NaCl nucleation from the melt, however, enthalpy has been proven to drive the the nucleation but $\overline{N}$ cannot do so [S11].

E. Discussions on electric force OPs

We further investigate the FES of simulations using the electric force OP $|F_{iW}|$ and $\overline{N}$ (Fig. S13). $\overline{N}$ is the local density descriptor and has been used to estimate the size of solid before [S11]. The shape of FES plots reproduces the same trend in plots of the last section: high $\overline{|F_{iW}|}$ ($\overline{|F_{iW}|} > 100\ kJ\ mol^{-1}\ nm^{-1}$) represents the liquid phase. As discussed above, high ion-water interaction leads to the dissolution of solid and forms liquid. For $\overline{|F_{iW}|} < 60$, at high thickness ($d = 1.9$ and 3.9 nm), it represents the solid phase with high $\overline{N}$. For $d = 1.2$ nm, low $\overline{|F_{iW}|}$ is also observed when $\overline{N} < 1$, the hydrated phase. Such observation supports the hypothesis that electric force, to be specific, the ion-water electric force, leads to the dissolution of the solid. Connected to the concept of dielectric constant discussed in the main text, the role of solvent is to screen the ion-ion electric force, a reduced dielectric constant for water slabs under confinement undermines the screening and leads to the formation of solid and hydrated phases.

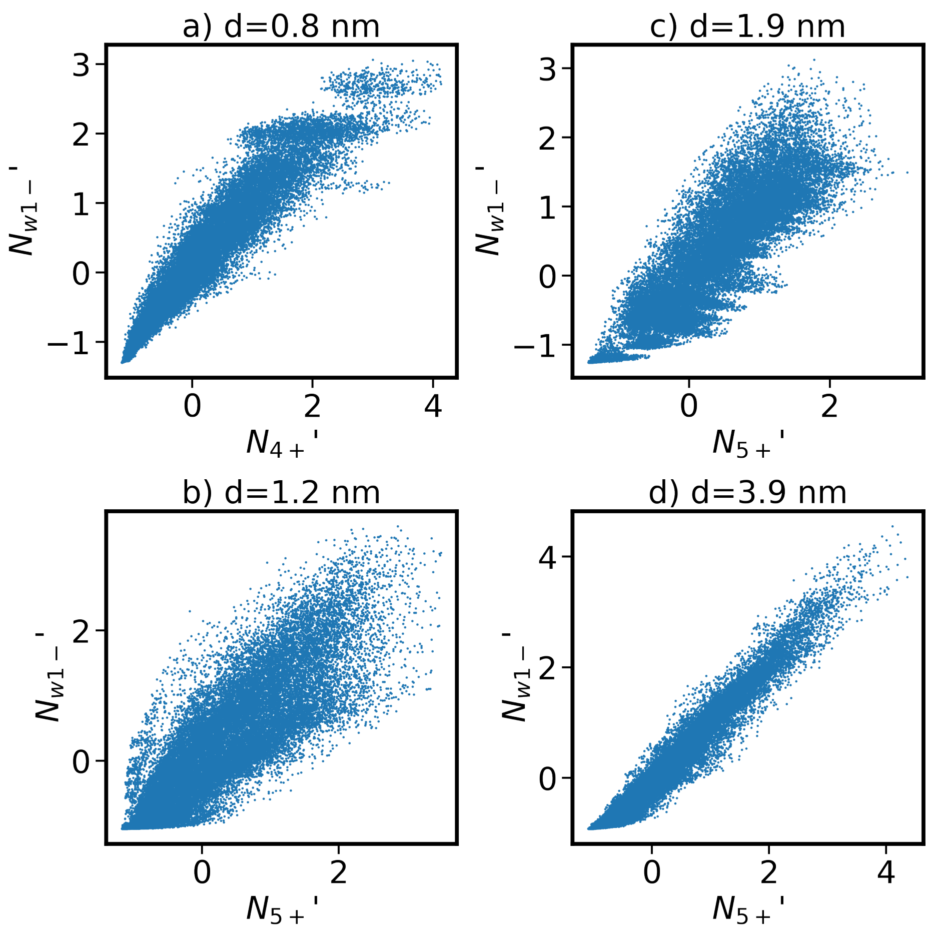

FIG. S9. The correlation between OPs N_{5+} vs. N_{W1-}, represented by the scatter plot.

F. Additional TERP results

TERP predicts a high score on Steinhardt OPs, especially $\overline{q_4}$, for $d = 3.9$ nm (Fig. 4 in the main text), consistent with our previous results [S11]. In bulk water, the ordered assembly of ions drives the nucleation because 1-step mechanism requires an ordered cluster [S11]. At other thicknesses, it is less important because these OPs are designed for three-dimensional space but not for the nano-confined water slab. Similar to $\overline{q_4}$ and $\overline{q_6}$, the number of ions with $CN > 5$, N_{5+}, represents the number of ions that belongs to the core of the solid phase in 3d space. In a confined water slab, there are not enough layers of ions, leading to a low score for $d = 1.2$ and 1.9 nm. For $d = 0.8$ nm, N_{4+} is the core for a completely 2d solid and it has a high score, instead of N_{5+} at other thickness. The total coulomb energy (U_E) of ions does not play an important role as the enthalpy does in nucleation from the melt. We speculate that interactions not included in this OP, such as the Lennard Jones and

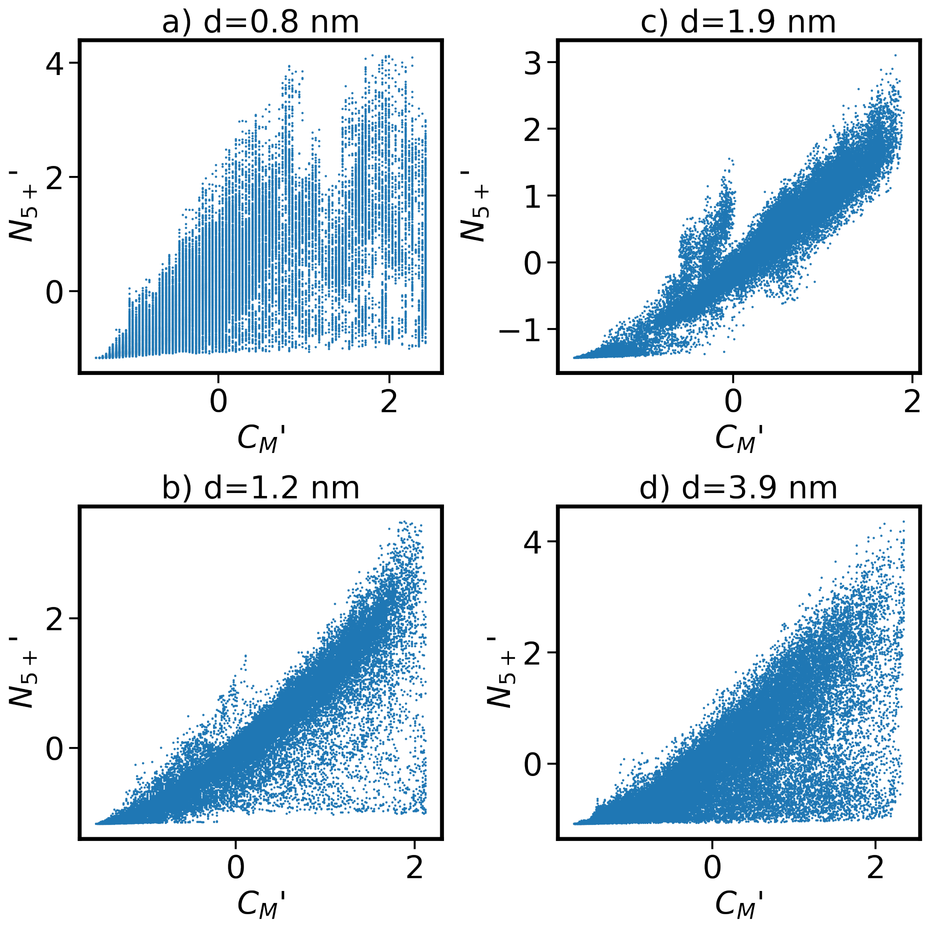

FIG. S10. The correlation between OPs C_M vs. N_{5+}, represented by the scatter plot.

ion-water interaction should not be overlooked and they may govern the nucleation process. The local density descriptor $\overline{N}$ does not have a high score in all simulations, consistent with our previous observations in bulk water [S11], though it can distinguish those crystal states and is often used in simulations of nucleation [S4, S20]. μ_N^2 represents the density fluctuation of the solution, making the homogeneous solution form high and low-density regions.

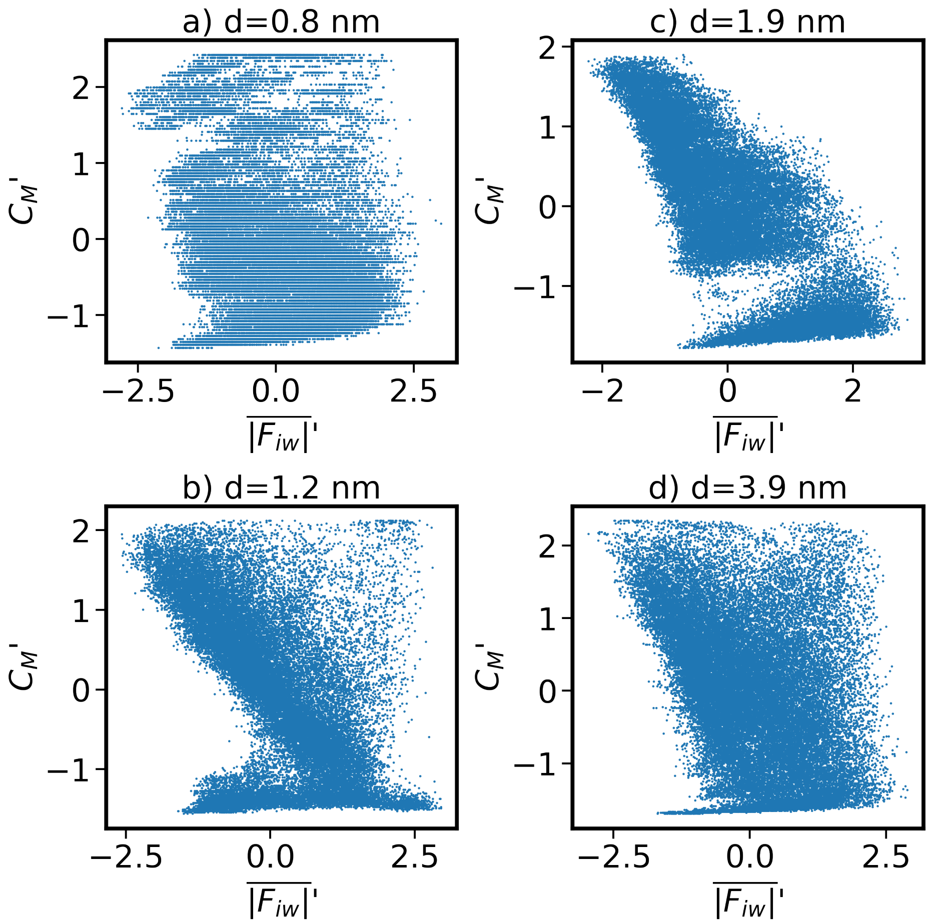

FIG. S11. The correlation between OPs $|F_{iW}|$ vs. C_M, represented by the scatter plot.

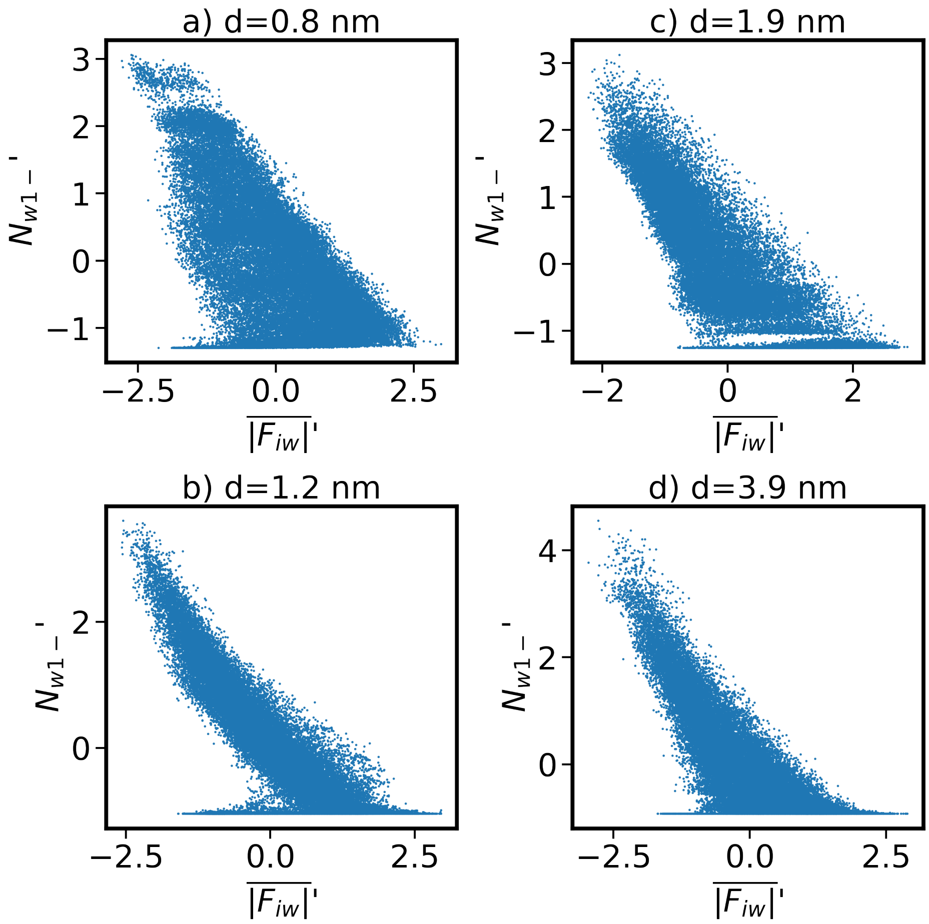

FIG. S12. The correlation between OPs $|F_{iW}|$ vs. N_{W1-}, represented by the scatter plot.

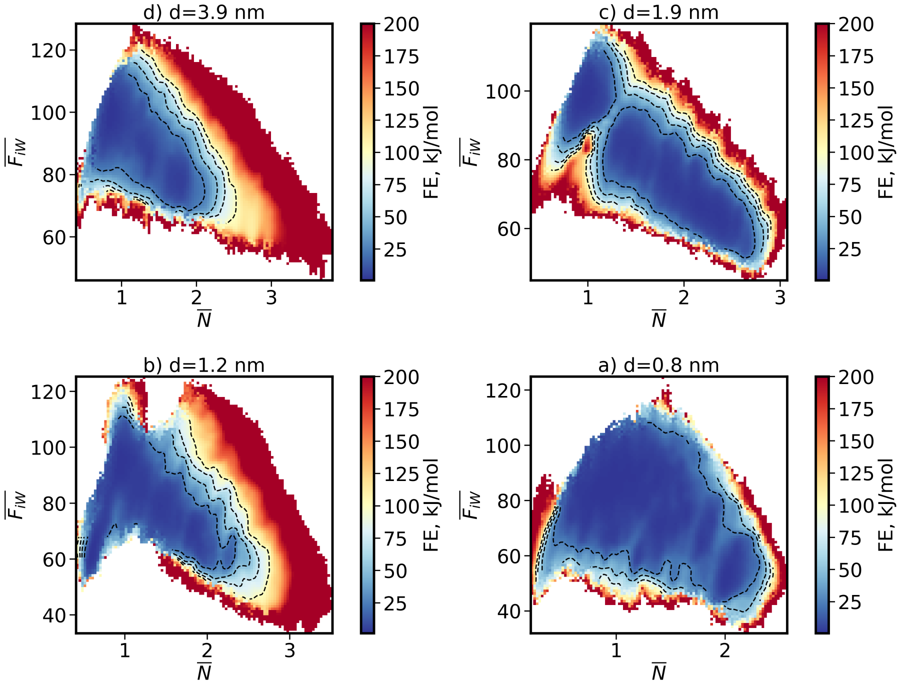

FIG. S13. The FES using $\overline{N}$ and $\overline{|F_{iW}|}$. The dash lines of the contour plots represent a free energy of 25 kJ/mol with a maxium of 100 kJ/mol. The unit for force is $kJ\ mol^{-1}\ nm^{-1}$.

[S1] D. Wang and P. Tiwary, State predictive information bottleneck, The Journal of Chemical Physics **154**, 134111 (2021).

[S2] E. R. Beyerle and P. Tiwary, Thermodynamically optimized machine-learned reaction coordinates for hydrophobic ligand dissociation, J. Phys. Chem. B 10.1021/acs.jpcb.3c08304 (2024).

[S3] S. Mehdi, D. Wang, S. Pant, and P. Tiwary, Accelerating all-atom simulations and gaining mechanistic understanding of biophysical systems through state predictive information bottleneck, Journal of Chemical Theory and Computation **18**, 3231 (2022).

[S4] Z. Zou, E. R. Beyerle, S.-T. Tsai, and P. Tiwary, Driving and characterizing nucleation of urea and glycine polymorphs in water, Proceedings of the National Academy of Sciences **120**, e2216099120 (2023).

[S5] D. Wang, R. Zhao, J. D. Weeks, and P. Tiwary, Influence of long-range forces on the transition states and dynamics of nacl ion-pair dissociation in water, J. Phys. Chem. B **126**, 545 (2022).

[S6] E. R. Beyerle, S. Mehdi, and P. Tiwary, Quantifying energetic and entropic pathways in molecular systems, The Journal of Physical Chemistry B **126**, 3950 (2022).

[S7] J. F. Dama, G. Rotskoff, M. Parrinello, and G. A. Voth, Transition-tempered metadynamics: Robust, convergent metadynamics via on-the-fly transition barrier estimation, J. Chem. Theory Comput. **10**, 3626 (2014).

[S8] D. Beckett and G. A. Voth, Unveiling the catalytic mechanism of gtp hydrolysis in microtubules, Proc. Natl. Acad. Sci. U S A **120**, e2305899120 (2023).

[S9] G. A. Tribello, M. Bonomi, D. Branduardi, C. Camilloni, and G. Bussi, Plumed 2: New feathers for an old bird, Computer Physics Communications **185**, 604 (2014).

[S10] P. Tiwary and M. Parrinello, A time-independent free energy estimator for metadynamics, The Journal of Physical Chemistry B **119**, 736 (2015).

[S11] R. Wang, S. Mehdi, Z. Zou, and P. Tiwary, Is the local ion density sufficient to drive nacl nucleation from the melt and aqueous solution?, J. Phys. Chem. B **128**, 1012 (2024).

[S12] S. Mehdi and P. Tiwary, Thermodynamics of interpretation, arXiv preprint , arXiv:2206.13475 (2022).

[S13] R. Wang, M. L. Klein, V. Carnevale, and E. Borguet, Investigations of water/oxide interfaces

by molecular dynamics simulations, WIREs Computational Molecular Science **11**, e1537 (2021).

[S14] D. Munoz-Santiburcio and D. Marx, Confinement-controlled aqueous chemistry within nanometric slit pores: Focus review, Chemical Reviews **121**, 6293 (2021).

[S15] P. Jungwirth and D. J. Tobias, Molecular structure of salt solutions: A new view of the interface with implications for heterogeneous atmospheric chemistry, J. Phys. Chem. B **105**, 10468 (2001).

[S16] P. Xu, R. Wang, H. Zhang, V. Carnevale, E. Borguet, and J. Suntivich, Cation modifies interfacial water structures on platinum during alkaline hydrogen electrocatalysis, J. Am. Chem. Soc. 10.1021/jacs.3c09128 (2024).

[S17] C. Zhang, S. Yue, A. Z. Panagiotopoulos, M. L. Klein, and X. Wu, Why dissolving salt in water decreases its dielectric permittivity, Phys. Rev. Lett. **131**, 076801 (2023).

[S18] A. R. Finney and M. Salvalaglio, A variational approach to assess reaction coordinates for two-step crystallization, The Journal of Chemical Physics **158**, 10.1063/5.0139842 (2023).

[S19] T. Karmakar, P. M. Piaggi, and M. Parrinello, Molecular dynamics simulations of crystal nucleation from solution at constant chemical potential, Journal of Chemical Theory and Computation **15**, 6923 (2019).

[S20] S.-T. Tsai, Z. Smith, and P. Tiwary, Reaction coordinates and rate constants for liquid droplet nucleation: Quantifying the interplay between driving force and memory, The Journal of Chemical Physics **151**, 154106 (2019).
